# Performance Evaluation and Fouling Propensity of Forward Osmosis (FO) Membrane for Reuse of Spent Dialysate

**DOI:** 10.3390/membranes10120438

**Published:** 2020-12-18

**Authors:** Chaeyeon Kim, Chulmin Lee, Soo Wan Kim, Chang Seong Kim, In S. Kim

**Affiliations:** 1 Global Desalination Research Center, School of Earth Sciences and Environmental Engineering, Gwangju Institute of Science and Technology (GIST), 123 Cheomdangwagi-ro, Buk-gu, Gwangju 61005, Korea; chaeyeon02@gist.ac.kr (C.K.); min90821@gist.ac.kr (C.L.); 2 Department of Internal Medicine, Chonnam National University Medical School, Gwangju 61469, Korea; skimw@chonnam.ac.kr (S.W.K.); laminion@daum.net (C.S.K.)

**Keywords:** hemodialysis, forward osmosis, spent dialysate, PA-TFC membrane, membrane fouling

## Abstract

The number of chronic renal disease patients has shown a significant increase in recent decades over the globe. Hemodialysis is the most commonly used treatment for renal replacement therapy (RRT) and dominates the global dialysis market. As one of the most water-consuming treatments in medical procedures, hemodialysis has room for improvement in reducing wastewater effluent. In this study, we investigated the technological feasibility of introducing the forward osmosis (FO) process for spent dialysate reuse. A 30 LMH of average water flux has been achieved using a commercial TFC membrane with high water permeability and salt removal. The water flux increased up to 23% with increasing flowrate from 100 mL/min to 500 mL/min. During 1 h spent dialysate treatment, the active layer facing feed solution (AL-FS) mode showed relatively higher flux stability with a 4–6 LMH of water flux reduction while the water flux decreased significantly at the active layer facing draw solution (AL-DS) mode with a 10–12 LMH reduction. In the pressure-assisted forward osmosis (PAFO) condition, high reverse salt flux was observed due to membrane deformation. During the membrane filtration process, scaling occurred due to the influence of polyvalent ions remaining on the membrane surface. Membrane fouling exacerbated the flux and was mainly caused by organic substances such as urea and creatinine. The results of this experiment provide an important basis for future research as a preliminary experiment for the introduction of the FO technique to hemodialysis.

## 1. Introduction

End-stage renal disease (ESRD) is a public health issue affecting more than 750 million people worldwide [1]. ESRD refers to a condition in which the kidney function is less than 10% of the normal kidney function and life-sustaining treatment such as medication or dietary control is not possible [2]. ESRD patients need renal replacement therapy (RRT) [3]. There are three representative methods in RRT, hemodialysis, peritoneal dialysis, and kidney transplantation, and the selection of treatment options is determined according to the patient’s condition [4].

Currently, peritoneal dialysis and hemodialysis are the most common options, and 63.2% of ESRD patients received hemodialysis, and only 7% received peritoneal dialysis [5,6]. The number of dialysis patients is on the rise with an annual increase of 8.7% [7]. Dialysis is a means clearance or removal of uremic toxins (such as urea, creatinine) and excess fluid from the blood and adding components deficient as needed [8]. The principle of hemodialysis involves the diffusion of solutes through a semipermeable membrane [9]. The diffusion occurs more actively with the greater difference in concentration and the larger the surface area of the membrane, and is affected by the pore size, thickness of the membrane, and the flow rate of blood and dialysate [10]. Hemodialysis uses counter-current flow, where concentrated dialysis solution flows in the opposite direction to the blood flow in the ultrafiltration (UF) process [11]. Counter-current flow serves to maximize the concentration gradient across the membrane and increase dialysis efficiency [12]. In this process, urea, creatinine, and other waste products, ions, etc. are diffused from the blood into the dialysis solution and removed. Hemodialysis takes at least four hours of dialysis treatment per time [13]. Patients receiving dialysis three times a week consume approximately 150 L of dialysate per session [14]. All over the world, the amount of water consumed during hemodialysis is 156 billion liters per year, and the amount of electric power consumed is 1.62 million kWh [15]. The wastewater created from hemodialysis treatment not only increases the cost burden in the post water treatment process but also causes water-related environmental problems. To address this issue, the membrane process that recycles spent dialysate effluent has been attracting much attention in and out of academia [16].

Membrane technology used in dialysis includes UF + reverse osmosis (RO) system and nanofiltration (NF) [17]. In hemodialysis, the RO system serves to supply purified water to dilute the concentrated dialysis solution used for the generation of concentration differences [18,19]. The purified water must be safe with chemical and microbial substances removed below established tolerance limits [20]. The RO system can exclude metal ions, aqueous salts, and molecules from the treated water [21]. But, the complexity of the RO system, high energy costs, membrane fouling/scaling, and large space requirements are major setbacks for the application of spent dialysate reuse [22]. In this regard, the forward osmosis (FO) system has recently been regarded as a promising alternative for conventional RO-based water purification system [23]. Since FO is an osmotically-driven membrane process, no external hydraulic pressure is required and the energy consumption is significantly lower than that of a conventional RO system [24,25]. Moreover, FO has superior flux stability against various types of membrane fouling issues [26], and the requirement of routine maintenance such as backwashing or chemical cleaning is insignificant compared to RO [27,28,29].

There are only a few studies that have been done on the application of FO for a spent dialysate reuse system. Most of these previous studies used cellulose triacetate (CTA) membrane with a smooth surface and fewer chemical bonds between membrane materials and inorganic deposits [16,22,30]. However, the thin-film composite (TFC) membrane has lower reverse salt transport and higher water flux, and better salt rejection than the CTA membrane [31]. In addition, compared to the CTA membrane, the TFC membrane has good pH stability and resistance to hydrolysis and biological degradation [32]. For the FO system to replace the conventional RO system, the TFC membrane with high water flux and selectivity are more suitable than the CTA membrane. In this study, we investigated the feasibility of FO in recovering and reusing purified water from the spent dialysate. The experiment was conducted using a polyamide thin-film composite (PA-TFC) membrane with high permeability and removal performance. We explored the effect of each condition on the membrane by adjusting the flow rate, additional pressure, and mode, etc. of the FO process. Accordingly, the correlation between the decrease in water flux over time, the reverse salt flux, and the rate of change in the ion concentration of the solution was confirmed. Finally, the physical property and the cause of membrane fouling were investigated.

## 2. Materials and Methods

### 2.1. Materials and Chemicals

A commercial flat sheet polyamide thin-film composite membrane (PA-TFC) was purchased from Toray Chemical Korea Inc. (Seoul, Korea). Sodium hydroxide (NaOH, ≥98.0%) and sodium dodecyl sulfate (SDS, ≥98.5% (GC)) were obtained from Sigma-Aldrich (Seoul, Korea). Spent dialysate and concentrated dialysis solution were kindly provided by kidney internal medicine at Chonnam National University Hospital (Gwangju, Korea). Concentrated dialysate (K-Bicart 761) was produced by Baxter, Seoul, Korea.

### 2.2. Analytical Methods

Field Emission Scanning Electron Scope (FE-SEM, JSM-7500F, Jeol, Tokyo, Japan) was used for analyzing the membrane surface. Ion Chromatography (IC, 930 COMPACT IC FLEX, Quantum Analytics, Oak Ridge North, TX, USA) was used to determine the concentration of cations and anions of feed and draw solutions before and after experiments. Electronic mass balance (GF-6100, A&D Company, Tokyo, Japan) was used to measure the variation in the weight of the draw solution to enable the calculation of the water flux. Total dissolved solids (TDS) meter (CON2700, Eutech Instruments, Singapore) was used to calculate reverse salt flux based on the conductivity change in the feed solution.

### 2.3. Characterization of the FO Membrane

**Figure 1:**
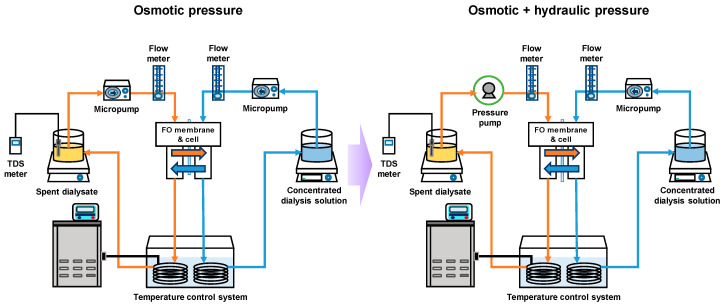
Schematic diagram of the FO & PAFO experiment system.

The basic FO performance of the membrane was estimated based on FO water flux (J_w_) and reverse salt flux (J_s_). A schematic diagram of the FO and pressure-assisted forward osmosis (PAFO) system is shown in Figure 1. The membrane was tested under the FO system with the active layer on the feed solution. The effectiveness of the FO membrane cell was 18.75 cm^2^ (2.5 cm × 7.5 cm). The membrane cell depth was 0.1 mm both active and the support layer.Also, the permeate spacer on the draw side FO membrane was employed for mechanical support. The flow rate of the feed and draw solutions was maintained constant at 300 mL/min. The temperature was maintained at 36.5 ± 0.5 °C. 1 M NaCl solution and deionized water (DI) were used to feed solutions and draw solutions.

### 2.4. Performance Test of the FO Process

The experiments were performed under cross-flow conditions. The membrane was stored in DI water at 4 °C before use. The membrane was tested under the FO process, with the active layer facing the feed solution (AL-FS) and the active layer facing the draw solution (AL-DS).

The tests were performed for 1 h using spent dialysate as feed solution and concentrated dialysis solution as a draw solution. The commercial concentrated dialysis solution used in the experiment is a high concentration solution containing the ions necessary for the patient. During hemodialysis, filtration is induced by extremely high osmotic pressure, supplying scarce ions to the patient. Table 1 shows the concentrated dialysis solution (K-Bicart 761) electrolytic concentration. The experimental temperature was maintained at 36.5 ± 0.5 °C to match the temperature of the patients. The operating conditions were conducted by adjusting the modes, flow rate, and pressure.

The flow rate was implemented at intervals of 100 mL/min within a range of at least 200 mL/min and a maximum of 500 mL/min. The pressure was applied on the feed side, and 0.5 bar, 1 bar, 1.5 bar, and the flow rate was kept constant at 500 mL/min. The variation in the concentration of feed solution was measured using the conductivity meter, and the change in weight of the draw solution was measured with balance. Samples were taken from the feed and draw solutions for the measurement of ion concentration.

To clean the pollutant remaining in the pipe after each experiment, chemical cleaning was performed for 30 min with 0.2 M NaOH and 0.2 M SDS solution to preserve the same condition as before. Then, the remaining chemicals were washed away using 20 L tap water and 4 L DI water.

### 2.5. Water Flux and Reverse Salt Flux

The water flux was calculated based on the volume changes in the permeate as a function of time (measurements at 1-min intervals), according to the following equation [33]:

(1) $$J_{w}=\frac{1}{A_{m}}\frac{∆V}{∆t}$$

where J*_w_* is the water flux at time *t*, *A_m_* is the effective membrane area (m^2^), *V* is the volume of permeate, and *t* is the time for permeate.

The reverse salt flux was determined by calculating the change of salt content in the feed solution as the following equation [34]:

(2) $$J_{s}=\frac{C_{f}V_{f}-C_{i}V_{i}}{A_{m}\times ∆t}$$

where J*_s_* is the reverse salt flux, *C_f_* and *C_i_* are the final and initial concentration of solute in the feed solution, and *V_f_* and *V_i_* are the final and initial volume of feed solution.

## 3. Results

### 3.1. Performance of the FO System

The FO performance test was performed using real patient spent dialysate and concentrated dialysis solution. The concentration of spent dialysate is about 7500 ppm and the concentrated dialysis solution is 101,000 ppm. The structure of the commercial Toray PA-TFC membrane used in the experiment consists of a selective active layer and porous support layer, the membrane thickness is 91.4 ± 1.3 μm, and porosity is 79.9 ± 2.3%. The pure water permeability is 8.818 L·m^2^·h/bar (LMH), and the membrane has a NaCl rejection of more than 97%.

**Figure 2:**
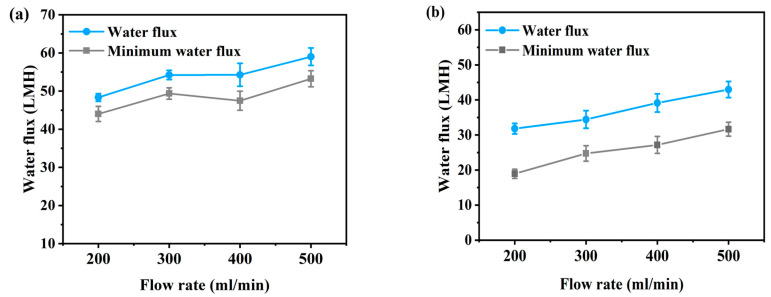
(**a**) Average water flux and minimum water flux of AL-FS mode and (**b**) average water flux and minimum water flux of AL-DS mode tested by osmotic pressure (FO). Feed solution: spent dialysate (1 L); Draw solution: concentrated dialysis solution (1 L); Operating time: 1 h; System temperature: 36.5 ± 0.5 °C.

**Figure 3:**
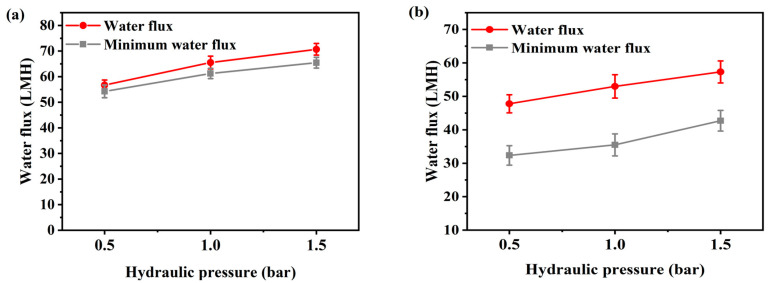
(**a**) Average water flux and minimum water flux of AL-FS mode and (**b**) average water flux and minimum water flux of AL-DS mode tested with hydraulic pressure (PAFO). Feed solution: spent dialysate (1 L); Draw solution: concentrated dialysis solution (1 L); Operating time: 1 h; Pressure: 0.5–1.5 bar; System temperature: 36.5 ± 0.5 °C. The experimental flow rate condition is fixed at 500 mL/min.

Figure 2 and Figure 3 present the flux according to flow rate and additional pressure in AL-FS mode and AL-DS mode. Water flux is the average value of the entire flux operating 1 h, and the minimum water flux is the average flux in the last 10 min. The temperature was maintained 36.5 ± 0.5 °C, similar to that of the patients, and the flow rate range of FO was set to 200–500 mL/min to match the conditions used for supplying concentrated dialysis solution in the hemodialysis. The feed solution flow rate is kept equal to the draw solution flow rate during all the experiments. In Figure 2, due to the increase in flow rate, FO water flux has increased from 48.3 LMH to 59 LMH (Figure 2a) and from 31.8 LMH to 43 LMH (Figure 2b), by approximately 11 LMH (23%). The flux change was most stable at a flow rate of 300 mL/min in AL-FS mode. In the same orientation, the flux of the cellulose triacetate (CTA) membrane was 18.6 LMH and the lowest flux of 200 mL/min in this experiment was 48.3 LMH [22]. Thus, it can be seen that sufficient water recovery flux has been reached at all flow rate conditions. Table 2 shows the performance of various methods for the reuse of spent dialysate. As shown in Figure 3, as the pressure increased, the water flux increased from 56.7 LMH to 70.7 LMH (Figure 3a) and from 47.8 LMH to 57.3 LMH (Figure 3b) about 13 LMH (23%). In contrast, the flux of the PAFO process, which added 0.5 bar of pressure at the same flow rate (500 mL/min), was 2.3 LMH lower than that of the FO process. This phenomenon is caused by an increase in the pressure difference between the supply inlet and the outlet. In other words, the supply pressure loss as a function of the hydraulic pressure applied from the draw solution when the crossflow of the feed solution is kept constant in PAFO mode [35], which can reduce water permeability.

The initial flux was higher in AL-DS mode, but the decrease in water flux overtime was nearly twice that of AL-FS mode, and the AL-DS mode was reduced more severe in the PAFO process. The flux in AL-DS mode decreased much rapidly as foulants accumulation and dilution of draw solution, which has been reported that the roughness characteristic of the membrane support layer tends to cause more pore-blocking than the active layer [36]. Besides the clogging of pores inside the membrane, the formation of a cake layer caused relatively severe membrane fouling in AL-DS mode [37,38]. Thus, fouling of the inner membrane, i.e., clogging of inner pores and adsorption of solutes occurred initial stage in AL-DS mode, resulting in rapid flux reduction [39].

Hemodialysis consumes 120 L of concentrated dialysis solution over 3–4 h, which represents a water flux of about 30–40 LMH. To be replaced by a spent dialysate reuse system, it is necessary to reach more than the water flux of current hemodialysis. According to the experimental results, the FO minimum water flux by flow rate is from 44 LMH to 53.2 LMH in AL-FS mode, and 18.9 LMH to 31.6 LMH in AL-DS mode, as shown in Figure 2. In Figure 3, the PAFO minimum water flux was higher than 30 LMH in both modes. Therefore, in the FO process, the AL-DS mode showed lower water flux than the hemodialysis system, in contrast, the AL-FS mode reached the target water flux and showed the higher efficiency.

### 3.2. Reverse Salt Flux

**Figure 4:**
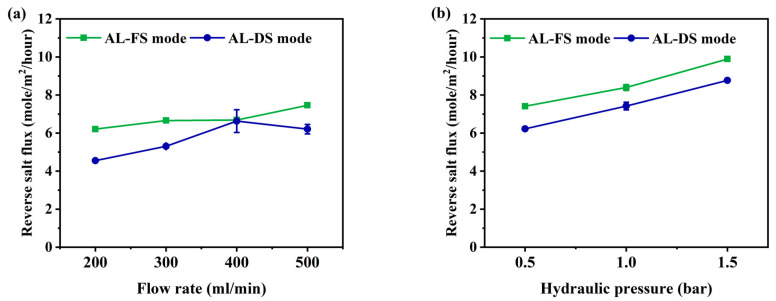
Comparison of the reverse salt flux under AL-FS and AL-DS mode. (**a**) FO process (flow rate: 200–500 mL/min); (**b**) PAFO process (0.5–1.5 bar), and experimental flow rate condition is fixed 500 mL/min; Operating time: 1 h; System temperature: 36.5 ± 0.5 °C.

**Figure 5:**
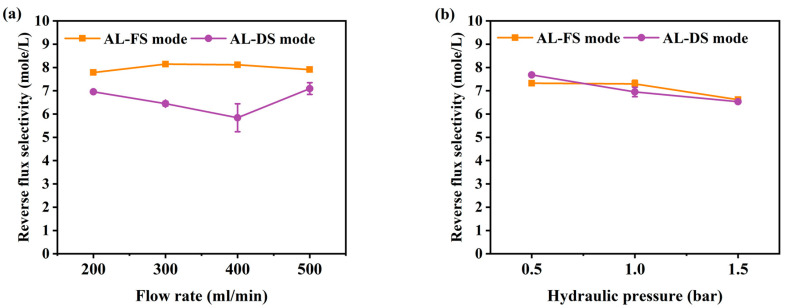
Comparison of the reverse flux selectivity under AL-FS and AL-DS mode. Calculated using the formula of J_w_/J_s_. (**a**) FO process (flow rate: 200–500 mL/min); (**b**) PAFO process (0.5–1.5 bar), and experimental flow rate condition is fixed 500 mL/min; Pressure was applied on the feed side. Operating time: 1 h; System temperature: 36.5 ± 0.5 °C.

The reverse salt flux (RSF) generally increases linearly with increasing water flux, depending on the selectivity and thermodynamic properties of the solution [42]. RSF not only reduces the driving force of the process but can also improve membrane fouling [43]. To understand membrane fouling, it is important to understand the mass transport of an osmotically driven membrane system [44]. The RSF performance of this experiment is shown in Figure 4. Due to the increase in flow rate, the RSF has increased from 6.2 to 7.5 mole/m^2^/h at AL-FS mode, and from 4.5 to 6.2 mole/m^2^/h at AL-DS mode (Figure 4a). The RSF increment by pressure was indicated to be from 7.4 to 9.8 mole/m^2^/h in AL-FS mode and 6.2 to 8.8 mole/m^2^/h in AL-DS mode (Figure 4b). In the PAFO condition, the membrane deforms under applied hydraulic pressure, resulting in higher RSF results than in the FO condition. In this paper, it was confirmed that the reverse salt flux also increased as the applied pressure increased [45]. In addition, the higher reverse salt flux of the TFC membrane in PAFO derives from the higher concentration difference across the membrane. Reverse flux selectivity (J_w_/J_s_) was computed to confirm the transport mechanism of water and salt under different two conditions, where J_w_ and J_s_ are the water flux and reverse salt flux, respectively [46]; The reverse flux selectivity of the AL-FS mode and AL-DS mode of the FO and PAFO processes under all experimental conditions are shown in Figure 5. The average value of AL-FS mode and AL-DS mode was 8 mole/L and 6.6 mole/L in the FO and 7.08 mole/L and 7.05 mole/L in the PAFO. As shown in Figure 5, the difference between the two modes under the FO condition was reduced in the PAFO condition. This is due to the result of the RSF of the PAFO condition (Figure 4b), which showed a steep rise compared to the RSF of the FO condition, which showed a similar increase (Figure 4a). When hydraulic pressure is applied to the feed side, the membrane active layer can deform significantly due to various compressive, stretch, and flexural stresses in the process [47]. Membrane deformation reduces the effective osmotic pressure gradient, resulting in low initial performance and poor mass transfer conditions as well as severe degradation of separation properties [45,48,49]. Also RSF increases as the membrane is compressed by a spacer in the direction of the support layer. Due to the characteristics of the flexible PA-TFC membrane, RSF showed a steadily increasing trend in proportion to the increasing pressure of the PAFO condition. Thus, the RSF result is more affected in the PAFO condition than in the FO condition. However, it is not necessary to consider the effect of reverse salt diffusion on the water flux in the experiment, and the subtle difference in reverse flux selectivity is predicted to be due to rapid membrane fouling over time.

### 3.3. The Transition of Ion Concentration in the Solution

The solution used as a feed in this experiment was spent dialysate from 5 to 6 anonymous patients, containing ion components of sodium (Na^+^), ammonium (NH_4_^+^), potassium (K^+^), calcium (Ca^2+^), fluoride (F^−^), chloride (Cl^−^), sulfate (SO_4_^2−^), and bicarbonate, and waste products such as urea and creatinine in different concentrations depending on the patient. In hemodialysis, urea and creatinine are filtered by the UF process and present in the spent dialysate. Urea is the primary metabolite derived from dietary protein and a circulating source of nitrogen-containing compounds. Creatinine is the product of muscle creatine catabolism. Both are relatively small molecules (60 and 113 daltons, respectively) that distribute throughout total body water [37]. Urea and creatinine are elements that need to be eliminated because kidney failure remains in the body without being filtered out of the blood, causing toxicity. The concentrated dialysis solution used as the draw solution was tested using the same product under all experimental conditions. The acid component of concentrated dialysis solution contains sodium chloride, sodium bicarbonate, potassium, magnesium, calcium, acetate (or citrate), and glucose.

**Figure 6:**
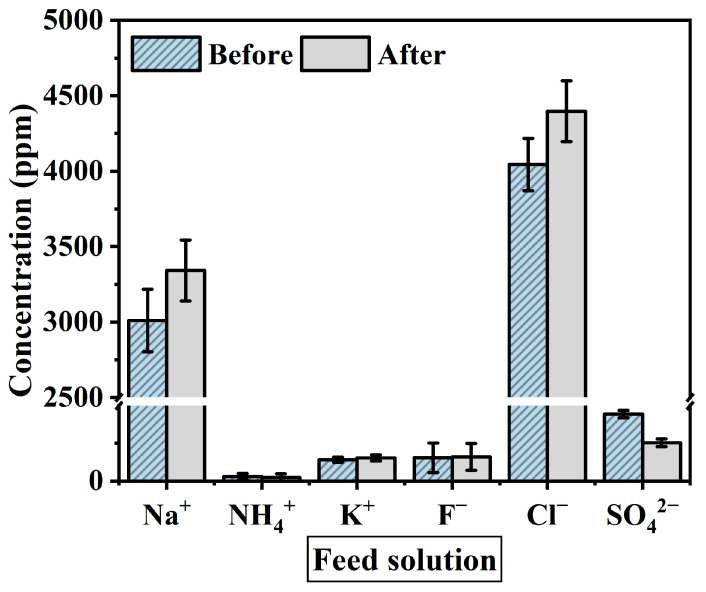
Average ion concentration of feed solution before and after the experiment. The experimental conditions are the FO process (flow rate: 200–500 mL/min) and PAFO process (0.5–1.5 bar) in AL-FS mode and AL-DS mode. Operating time: 1 h; System temperature: 36.5 ± 0.5 °C.

**Figure 7:**
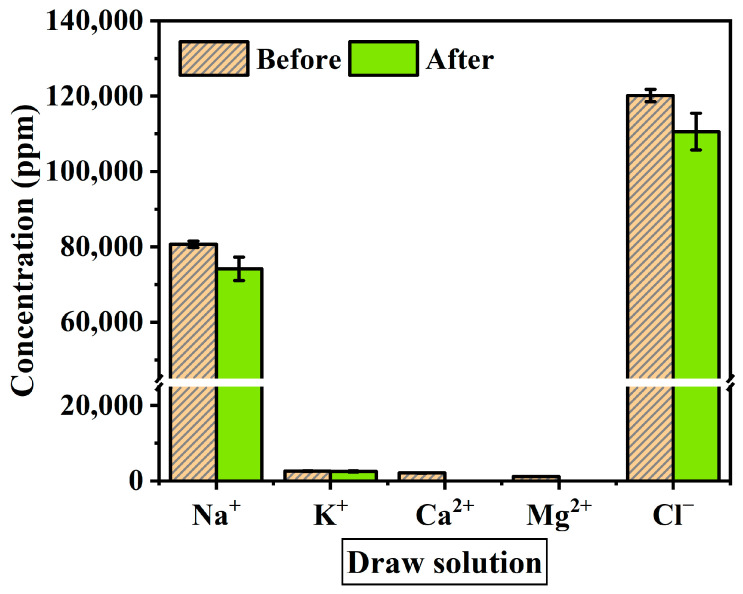
Average ion concentration of draw solution before and after the experiment. The experimental conditions are the FO process (flow rate: 200–500 mL/min) and PAFO process (0.5–1.5 bar) in AL-FS mode and AL-DS mode. Operating time: 1 h; System temperature: 36.5 ± 0.5 °C.

When materializing a spent dialysate reuse system, ion exchange through the membrane and changes in ion concentration are factors to consider as they affect the flux reduction. The solution before and after the experiment was measured with IC to identify the change in ion concentration during the experiment. The ions used as standard solutions are Na^+^, NH_4_^+^, K^+^, Ca^2+^, Mg^2+^, F^−^, NO_2_^−^, Cl^−^, Br^−^, NO_3_^−^, PO_4_^3−^ and SO_4_^2−^. Figure 6 shows the average value of the ion concentration in feed solution before and after the FO membrane filtration experiment (1 h). Figure 7 presents ion concentration in draw solution under the same condition. Both solutions consisted mostly of Na^+^ and Cl^−^ ions. As shown in Figure 7, ion concentrations after the experiment decreased by Na^+^ 6521 ppm, K^+^ 108.2 ppm, and Cl^−^ 9593.4 ppm, and the Mg^2+^ and Ca^2+^ concentration in the draw solution were not measured after the experiment. On the other hand, after the experiment in feed solution tends to increase by K^+^ 12.2 ppm, F^−^ 4.9 ppm, Na^+^ 331.6 ppm, and Cl^−^ 353.4 ppm (Figure 6). However, NH_4_^+^ and SO_4_^2−^ concentrations were decreased for 5 ppm and 20 ppm respectively. This means that polyvalent ions such as NH_4_^+^ and SO_4_^2−^ have been rejected while monovalent ions were passed through the membrane in the principle of molecular sieve effect and reverse solute diffusion. The average feed solution concentration increased by 7500 ppm to 8600 ppm and the draw solution concentration decreased by 101,000 ppm to 97,160 ppm after the experiment. Since the feed solution is concentrated and discarded after recovering a certain amount of clean water, the concentration of the feed solution only affects the recovery rate of the system, which changes due to the difference in concentration. However, the draw solution is provided directly to the patient through a reuse system, the concentration of ions must be considered important. In the draw solution, K^+^ showed the lowest rejection, and Cl^−^ showed the highest rejection (Figure 7). Although most patients do not have a significant difference in serum potassium after hemodialysis, patients with diseases such as arrhythmia may get side effects such as hypokalemia [50,51]. As shown in the experimental results, a decrease in the K^+^ concentration of concentrated dialysis solution does not affect most patients, but some patients should be used separately. Also, the membrane removal of NH_4_^+^, which is known to be more toxin than urea, is a positive outcome in the process of developing a spent dialysate reuse system [37,52].

After the experiment, the causes of the reduction in ion concentration of NH_4_^+^, SO_4_^2−^, Ca^2+^, and Mg^2+^ are expected by two factors: (1) The ions remained in the solution at a low concentration that could not be measured; (2) The ions were filtered by the membrane and remained on the membrane surface. Among these ions, Ca^2+^ and Mg^2+^ are expected to remain mainly on the membrane surface and cause scaling, which can be confirmed through SEM analysis. According to a previous study, the Ca^2+^ concentration in the concentrated dialysis solution is not very important since the temporary change in blood Ca^2+^ concentration during dialysis treatment does not affect the patient’s health significantly [53]. Also, Ca^2+^ can be dosed through a concentrated dialysate solution in case of calcium deficiency.

### 3.4. Flux Comparison of Baseline Flux and Long-Term Experiment

**Figure 8:**
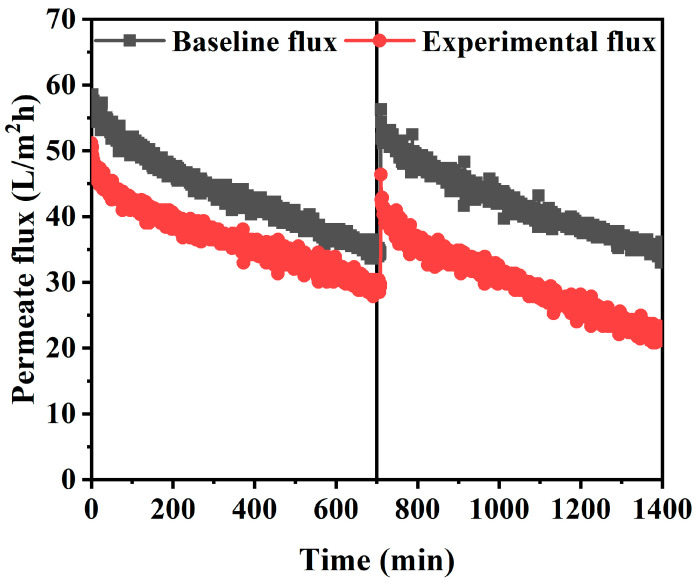
FO experiment with flow rate condition of 300 mL/min in AL-FS mode. Feed solution of experimental baseline flux: NaCl (7500 ppm); Feed solution of experimental water flux: spent dialysate (7500 ppm); Draw (baseline and experimental flux): concentrated dialysis solution (101000 ppm); Operating time: 24 h; System temperature: 36.5 ± 0.5 °C.

During the long-term FO experiment, the reduction of the effective osmotic pressure gradient across the FO membrane and the additional hydraulic resistance of the membrane due to membrane fouling led to the flux decline. To evaluate the effects of both main causes, the FO baseline flux was measured by using a feed solution with 7500 ppm of NaCl concentration, which is equivalent to the average TDS concentration of spent dialysate. In both experiments, the identical concentrated dialysis solution was used as the draw solution. The FO experiment was conducted for 24 h by determining a flow rate of 300 mL/min under the AL-FS mode condition, which was estimated to be most suitable for a spent dialysate reuse system. In Figure 8, the experimental FO flux was 53 LMH from the initial of the experiment, slightly lower than the baseline FO flux of 58 LMH. The difference is predicted to be due to rapid membrane fouling by inorganic ions and organic substances contained in the solutions. As evidence for that, experiments with artificially made spent dialysate have shown that the effective osmotic pressure gradient decline is the main cause of the flux reduction [22]. Considering that the spent dialysate used in this experiment is from the real hemodialysis patients, it can be seen that the flux decrease in Figure 8 is due to membrane fouling by organic matters. In the baseline FO experiment, fouling does not occur because there are no organic substances such as urea and creatinine in the feed solution. After 12 h, the baseline flux was 56.3 LMH, 1.7 LMH lower than the initial baseline flux as shown in Figure 8. This is forecasted to be due to membrane scaling by Ca^2+^ and Mg^2+^ contained in the draw solution. However, the water flux estimated to have been reduced by scaling is negligible so the main origin of flux reduction in the baseline FO experiment is the decrease in osmotic pressure.

Moreover, the internal concentration polarization (ICP) of porous membrane support, one of the major drawbacks of FO, may be considered as a cause of the decrease in flux [38]. Thus, the effective driving force of the FO process (difference in concentration across the dense active layer) is much lower than the apparent driving force (difference in feed solution concentration and draw solution concentration) [38]. The TFC membrane used in the experiment had the same properties as the substrate, and the flux reduction would have occurred due to the ICP generated in the porous support layer. Also, the concentrated dialysis solution used as the draw solution is a very high concentration solution and can cause severe ICP due to reverse salt diffusion. The mass transfer of the support layer cannot be directly enhanced by cross-flow, so ICP has a significant impact on FO performance and plays a major role in FO separation [54]. However, compared to the baseline experiment, the experimental flux using a solution containing more fouling shows a relatively stable flux reduction trend in Figure 8. Most of the TFC membranes operated in the FO process (AL-FS mode) mitigate irreversible pore blocking caused by the penetration of fouling into the porous substrate [55]. Furthermore, due to the ICP self-compensating effect, FO essentially has excellent flux stability against fouling [56]. Attempts to reduce the flux of the membrane are compensated for by reducing the ICP [57].

### 3.5. Analysis of the Membrane Fouling

**Figure 9:**
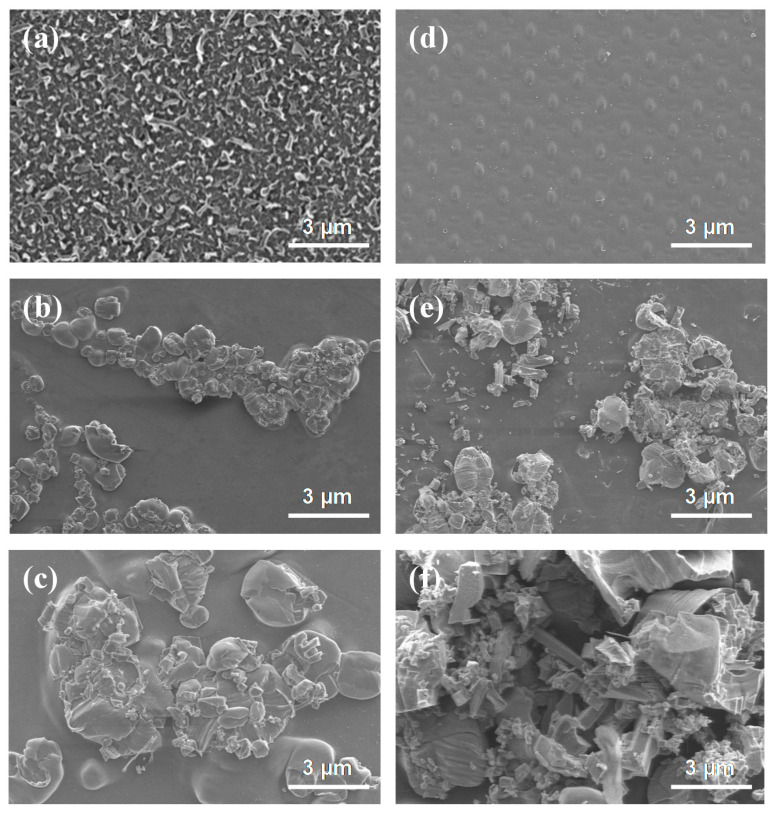
SEM images of the (**a**) active layer of pristine membrane, (**d**) support layer of pristine membrane, (**b**,**c**) active layer of fouled membrane, and (**e**,**f**) support layer of the fouled membrane. The fouled membrane was produced after 24 h experiment. FO process (in AL-FS mode); Flow rate: 300 mL/min; Temperature: 36.5 ± 0.5 °C.

Membrane fouling is typically classified into biofouling, organic fouling, and inorganic fouling [56]. To identify the cause of membrane fouling in this experiment, SEM images of the pristine membrane and the fouled membrane were observed. As shown in Figure 9a, the membrane surface of the pristine membrane of the active layer. Figure 9d is the membrane surface of the support layer of the pristine membrane. The fouling experiment was performed 24 h in the FO process in AL-FS mode with a flow rate of 300 mL/min using spent dialysate. The ions contained in the solution used in FO are shown in Figure 6 and Figure 7. SEM images show that the membrane surface is covered with a layer of foulants on active and support layers after the long-term experiment.

The fouling layer that formed during FO long-term experiment was compact and densely packed with inorganic foulants in Figure 9c,e. As a substance that can cause inorganic fouling, NH_4_^+^ and SO_4_^2−^ are composed in the feed solution, and Ca^2+^ and Mg^2+^ are included in the draw solution. Polyvalent ions such as Ca^2+^ and Mg^2+^ can reinforce the fouling layer because they can act as cross-linking ions between the protein fouling layer and the hydrophilic side chains of dissolved proteins, peptides, and phospholipids [58,59]. This will create a denser layer of fouling, and the solution chemistry effect of monovalent ions is not significant [60]. In addition, alkaline cation ions such as Ca^2+^ and Mg^2+^ ions caused more fouling problems in the membrane when combined with polyanions such as SO_4_^2−^ [61].

The membrane scaling reduces the effective surface area of the membrane, causing additional resistance to flow and mass transfer, which can be exacerbated by membrane fouling in the presence of organic matters [62]. Membrane scaling occurs when the concentration of sparingly soluble salts in the feed solution reaches supersaturation and the salts crystallize directly on the membrane surface or crystallize in bulk solution and deposit on the membrane surface [57]. As shown in Figure 9f, the fouling of the support layer was confirmed to be accumulated on the membrane by crystallization of ions contained in the draw solution, and it is due to Ca^2+^ and Mg^2+^ not measured in the draw solution after the experiment. Thus, Ca^2+^ and Mg^2+^ are the main substances that cause organic fouling, and, during long-term FO experiments, they were adsorbed on the membrane surface and caused scaling.

In forward osmosis, membrane fouling has been shown to be affected by the composition of the feed and draw solutions, which is due to the mechanism of reverse salt diffusion enhanced organic substances [63]. During filtration, some protein molecules can penetrate the membrane and cause internal fouling, preventing pores. Protein has been validated by researchers as organic substances that cause fouling in the membrane separation process [64], and organic substances such as urea and creatinine, which contain protein molecules produced in the human body in the spent dialysate, will be significantly related. As shown in Figure 9b,c, the membrane surface of the FO experiment using spent dialysate composed of organic substances and various types of inorganic ions as feed solution showed the most severe fouling. It also showed a similar shape to the SEM images of urea and creatinine presented in previous studies and was estimated to remain on the surface of the active layer after being removed by the membrane (Figure 9b).

SEM analysis confirmed that the low flux of the FO experiment compared to the baseline flux in Figure 8 was caused by membrane fouling (i.e., inorganic fouling, organic fouling, scaling) with a decrease in the effective osmotic pressure gradient. Membrane fouling is affected by complex phenomena such as initial adsorption, precipitation, and gel formation, the interaction between solutes, membrane properties, and operating conditions [65]. The experiment under various operating conditions (i.e., flow rate in the range of 200–500 mL/min, pressure in the range of 0.5–1.5 bar, AL-FS, and AL-DS modes) has important consequences in determining the cause of membrane fouling.

## 4. Conclusions

In this study, we investigated the feasibility of introducing the FO process into a spent dialysate reuse system. The water flux required for hemodialysis was reached in all experimental conditions, and the AL-FS mode showed a relatively stable flux tendency over time. The 300 mL/min of the FO process (in AL-FS mode) with the least flux decline after 1 h compared to the average water flux is decided to be suitable for the spent dialysate reuse system. The RSF was increased by the membrane deformation in the PAFO process, increasing the salt concentration across the membrane. The flux decline was exacerbated by membrane fouling. Inorganic fouling was mostly affected by polyvalent ions in the solution, and organic fouling was caused by organic substances bound to protein molecules such as urea and creatinine. Urea and creatinine have been partially removed from the membrane, and a high removal rate of these toxic substances was required for the reuse of spent dialysate. In addition, Ca^2+^ and Mg^2+^ were removed by the membrane during filtration and remained on the surface, causing membrane scaling. The result of the FO experiment was that the purified water required for hemodialysis was sufficiently recovered and was relatively stable in reducing the amount of spent dialysate. Significant water savings compared to conventional hemodialysis process in which a commercial concentrated dialysis solution is diluted with a large amount of purified water. Our research provides a new foundation for the possibility of developing a reuse system for FO-based spent dialysate. It is recommended to use a new membrane for safety.

## Figures and Tables

**Table 1:** A concentrated dialysis solution was used in this study.

The Electrolytic Concentration of Concentrated Dialysis Solution (K-Bicart)	
Components	Concentration
Na^+^	140 (mEq/L)
K^+^	2.0 (mEq/L)
Ca^2+^	2.5 (mEq/L)
Mg^2+^	1.0 (mEq/L)
Cl^−^	108.5 (mEq/L)
Ac^−^	3.0 (mEq/L)
HCO_3_^−^	34 (mEq/L)
Glucose (C_6_H_12_O_6_)	1 g/L

**Table 2:** Comparison of the performance of different methods for reuse of spent dialysate.

Membrane	Used Solutions	Results of Performance	Summary	Reference
Commercial flat sheet thin-film composite (TFC) membrane	Actual spent dialysate from patients as the feed solution and concentrated dialysis solution as the draw solution	With increasing flow rate, the FO average water flux has increased from 48 to 59 LMH in AL-FS mode and from 31 to 43 LMH in AL-DS mode.	Experimental conditions: AL-FS mode/AL-DS mode; under the FO and PAFO process; a range of flow rate (200–500 mL/min); temperature: 36.5 ± 0.5 °C; operating time: 1 h	In this study
Commercial flat sheet triacetate (FTS-CTA) membrane	Synthetic spent dialysate (SSD) as the feed solution and dialysis concentrate as the draw solution	The FO flux declined from 18.6 to 4.4 L/m^2^ h and water recovery of 64%.	Cross-flow velocity of 25 cm/sec at 10 bar; temperature: 25 ± 0.5 °C; flow rate was kept constant at 900 mL/min	[22]
Commercial FTS-CTA membrane and tailor-made hollow fiber thin-film composite (HF-TFC) membrane	Deionized water as the feed solution and dialysis concentrate as the draw solution	The initial flux of the HF-TFC membrane (33.5 LMH) and CTA membrane (17.6 LMH).	Pressure was applied 5–14 bar (CTA membrane) and 2–5 bar (HF-TFC membrane); The cross-flow rates of solutions kept constant at 0.9 L/min (flow velocity of 0.25 m/s) for CTA membrane and 0.2, 1.8 L/min for HF-TFC membrane; temperature: 25 ± 0.5 °C	[16]
Two-dimensional transition-metal carbide (MXene)	Simulated dialysate and aqueous solution	Adsorption rate with higher adsorption capacity (45.7 and 17.0 mg/g for creatinine and uric acid).	Higher adsorption capacity compared to activated carbon due to the open interlayer structure and hydrophilic surface termination of Ti_3_C_2_T_x_	[40]
Graphene oxide (GO) nanosheet	Urea solution	Urease was removed of 2194 ± 110, 1604 ± 90, 1172 ± 59 and 605 ± 30 mg/g for 80, 60, 40 and 20 mg/dL urea solutions.	There is no negative effect, it shows good blood compatibility, and the urea rejection is about 80% for urea solution.	[41]
